# The Mechanism Actions of Astragaloside IV Prevents the Progression of Hypertensive Heart Disease Based on Network Pharmacology and Experimental Pharmacology

**DOI:** 10.3389/fphar.2021.755653

**Published:** 2021-11-05

**Authors:** Haoran Jing, Rongsheng Xie, Yu Bai, Yuchen Duan, Chongyang Sun, Ye Wang, Rongyi Cao, Zaisheng Ling, Xiufen Qu

**Affiliations:** ^1^ Department of Cardiovascular, the First Affiliated Hospital of Harbin Medical University, Harbin, China; ^2^ Department of CT, the First Affiliated Hospital of Harbin Medical University, Harbin, China; ^3^ Blood Transfusion Department, the First Affiliated Hospital of Harbin Medical University, Harbin, China; ^4^ Department of CT, the Second Affiliated Hospital of Harbin Medical University, Harbin, China

**Keywords:** cardiac damage, astragaloside IV, hypertensive heart disease, network pharmacology, inflammation, antioxidant

## Abstract

Astragaloside IV (AS-IV) has been used to treat cardiovascular disease. However, whether AS-IV exerts a protective effect against hypertensive heart disease has not been investigated. This study aimed to investigate the antihypertensive and cardioprotective effects of AS-IV on L-NAME-induced hypertensive rats via network pharmacology and experimental pharmacology. The network pharmacology and bioinformatics analyses were performed to obtain the potential targets of AS-IV and hypertensive heart disease. The rat hypertension model was established by administrated 50 mg/kg/day of L-NAME for 5 weeks. Meanwhile, hypertension rats were intragastrically administrated with vehicle or AS-IV or fosinopril for 5 weeks. Cardiovascular parameters (systolic blood pressure, diastolic blood pressure, mean arterial pressure, heart rates, and body weight), cardiac function parameters (LVEDd, LVEDs, and fractional shortening), cardiac marker enzymes (creatine kinase, CK-MB, and lactate dehydrogenase), cardiac hypertrophy markers (atrial natriuretic peptide and brain natriuretic peptide), endothelial function biomarkers (nitric oxide and eNOS), inflammation biomarkers (IL-6 and TNF-α) and oxidative stress biomarkers (SOD, MDA, and GSH) were measured and cardiac tissue histology performed. Network pharmacological analysis screened the top 20 key genes in the treatment of hypertensive heart disease treated with AS-IV. Besides, AS-IV exerted a beneficial effect on cardiovascular and cardiac function parameters. Moreover, AS-IV alleviated cardiac hypertrophy via down-regulating the expression of ANP and BNP and improved histopathology changes of cardiac tissue. AS-IV improved endothelial function via the up-regulation of eNOS expression, alleviated oxidative stress via increasing antioxidant enzymes activities, and inhibited cardiac inflammation via down-regulating IL-6 and TNF-α expression. Our findings suggested that AS-IV is a potential therapeutic drug to improve L-NAME-induced hypertensive heart disease partly mediated via modulation of eNOS and oxidative stress.

## Introduction

Hypertension is a common cardiovascular disease and a primary contributory factor for pathological cardiac dysfunction and remodeling, which seriously harms the structure and function of the heart (Santos and Shah, 2014). Besides, persistent hypertension may cause fibrosis and left ventricle hypertrophy, which even resulting in heart failure and renal injury (Gradman and Alfayoumi, 2006; Uraizee et al., 2013). Loss of nitric oxide (NO) bioavailability and deficiency in endogenous NO synthesis are thought to underlie functional and histological cardiac injury during this process (Yang et al., 2015). Abnormal changes in NO bioavailability or synthesis evokes endothelial dysfunction, which is also related to the progression of diabetes, heart failure, and hypertension (Moncada, 1992). It has been reported that NO exerts the cardioprotective effect by alleviating cardiac apoptosis and remodeling after myocardial infarction via inhibition of oxidative stress (Smith et al., 2005). Moreover, Up-regulation of eNOS expression declined fructose-evoked insulin resistance and hypertension in rats (C. X. Zhao et al., 2009). The pathogenesis of hypertension involves complex interplays of pathophysiologic, environmental, and genetic factors. Oxidative stress plays a vital role in the pathophysiologic process of hypertension (Touyz et al., 2020). It contributes to renal injury and vascular dysfunction associated with hypertension (Small et al., 2018). Besides, oxidative stress could decrease the bioavailability of NO, leading to vasoconstriction, which could even cause hypertension (Harrison et al., 2003). The increasing evidence indicated that inflammatory cytokines, including IL-6, TNF-α, IFN-γ, and IL-17 secreted from T cells, contributed to both vascular and renal injury and dysfunction, causing organ injury, high blood pressure, and oxidative stress (McMaster et al., 2015; Zimmer et al., 2020). N^ω^-nitro-L-arginine methyl ester (L-NAME), a nitric oxide synthase inhibitor, obviously causes NO deficiency and evokes high blood pressure in an animal model (Biwer et al., 2013). Chronic administration of L-NAME could cause cardiac hypertrophy *via* up-regulation of brain natriuretic peptide (BNP) and atrial natriuretic peptide (ANP) *in vivo* (Suo et al., 2002). Treating rats with L-NAME could induce vascular endothelial injury, and this animal model is widely used in the study of cardiovascular and hypertension diseases (Ribeiro et al., 1992). Additionally, declined antioxidant defense systems and increased production of reactive oxygen species are present in L-NAME-induced hypertensive rat model (Rincón et al., 2015; Zambrano et al., 2013). Therefore, developing new active ingredients with antioxidant effects that could improve endothelial function, and reduce oxidative stress and inflammation might be beneficial for preventing and treating hypertensive heart disease.

Astragaloside IV (AS-IV) is a major active compound of *Astragalus membranaceus.* It has been useful in the treatment of nonalcoholic fatty liver disease via regulating inflammatory cytokines (Liu et al., 2020). Additionally, it has been reported that AS-IV could decrease obesity-associated hypertension via improving leptin resistance and suppressing inflammatory reactions (Jiang et al., 2018). AS-IV could alleviate cardiac hypertrophy and improve cardiac function via activating Nrf2 (Nie et al., 2019). However, the effects of AS-IV against hypertension-associated cardiac damage are poorly investigated. Thus, the antihypertensive and cardioprotective effects of AS-IV were explored in the L-NAME-evoked hypertensive model, and the underlying mechanism actions of protection effects were evaluated by measuring oxidative stress and endothelial dysfunction-related biomarkers.

## Materials and Methods

### Prediction of AS-IV-Associated Targets

The CTD database (http://ctdbase.org/), PubChem database (https://pubchem.ncbi.nlm.nih.gov), and Swiss Target Prediction database (http://www.swisstargetprediction.ch/) were used to identify potential targets of AS-IV.

### Prediction of Hypertensive Heart Disease-Associated Targets

The CTD database (http://ctdbase.org/) and Genecards database (https://www.genecards.org/) were used to identify potential targets of hypertensive heart disease. The hypertensive heart disease-associated targets were obtained by searching the keyword “hypertensive heart disease” in these databases.

Construction of protein-protein interaction (PPI) network and core genes identification.

A Venny2.1.0 tool was used to collect the common targets of the AS-IV and hypertensive heart disease. Then, the PPI network of these common targets was constructed using the STRING database (https://stringdb.org/). Then, Cytoscape software (www.cytoscape.org/) was used to visualize and integrate the topological parameters of common targets in the PPI network. The degree of each protein node was calculated using the CytoHubba plugin. Then, the top 20 genes were identified as core genes.

### Enrichment Analysis and Construction of the Compound-Targets-Pathways-Disease Network

KEGG pathway analysis was performed using the Metascape platform (http://metascape.org/gp/#/main/step1) to obtain the AS-IV-mediated pathways against hypertensive heart disease. Cytoscape software (www.cytoscape.org/) was used to construct a compound-targets-pathways-disease network based on the results of PPI and KEGG analysis.

### Experimental Verification

#### Animal Experimental Protocol

Male Sprague-Dawley rats (6–8 weeks old and 180–220 g) were purchased from the animal center of Harbin Medical University and housed under temperature- and humidity-controlled animal room, with 12 h light-dark cycles and free access to food and water. All animal experiments were performed by National Institutes of Health guidelines and approved by the Animal Ethics Committee of the First Affiliated Hospital of Harbin Medical University.

After 1 week of acclimation, all animals were randomly divided into the five groups (*n* = 8 for each group) and treated as follows: Control group (CON), rats only received carboxymethyl cellulose solution (1%) daily; CON + HAS-IV group, rats in the CON group received a high dose of AS-IV (40 mg/kg) daily; L-NAME group (LN), rats received 50 mg/kg of L-NAME in carboxymethyl cellulose solution (1%) daily for 5 weeks to evoke hypertension (Berkban et al., 2015); LN + LAS-IV group, rats in the LN group received a low dose of AS-IV (20 mg/kg) daily; LN + HAS-IV group, rats in the LN group received a high dose of AS-IV (40 mg/kg) daily. LN + fosinopril group, rats in the LN group received fosinopril (4.67 mg/kg) daily. The doses of AS-IV and fosinopril were selected according to the previous report (Jiang et al., 2018; Wang et al., 2020). Drugs were suspended in carboxymethyl cellulose solution (1%). Carboxymethyl cellulose solution (1%) or drugs were intragastrically administered daily for 5 weeks.

### Measurement of Cardiovascular Parameters

After 5 weeks of continuous administration, a non-invasive blood pressure measurement and analysis system (ALC-NIBP, ALCBIO, China) were used to measure the systolic blood pressure (SBP), diastolic blood pressure (DBP), mean arterial pressure (MAP), and heart rates (HR) in conscious rats based on manufacturer’s instruction.

### Assessment of Cardiac Function

At the end of the experiment, the rats were fasted for 18 h and anesthetized with 40 mg/kg of sodium pentobarbital by intraperitoneal injection. The Doppler echocardiography (Agilent Sonos5500) was used to measure the left ventricular end-diastolic dimension (LVEDd), left ventricular end-systolic dimension (LVEDs), and fractional shortening (FS) of each group.

### Collection of Tissue and Blood Samples

At the end of the experiment, rats were anesthetized with 40 mg/kg of sodium pentobarbital by intraperitoneal injection and euthanized by inhaling CO_2_. Then, the blood samples were rapidly collected from the abdominal aorta. Hearts and thoracic aortas were rapidly harvested and stored at −20°C for further analysis.

### Measurement of Cardiac Marker Enzymes Activities

Collected blood samples were centrifuged at 3,000 r/min for 15 min at 4°C and the serum was obtained. The activities of creatine kinase (CK), creatine kinase-MB (CK-MB), and lactate dehydrogenase (LDH) were measured using commercially available kits (Jiancheng Bioengineering, Nanjing, China) based on the manufacturer’s instruction.

### Assay of Endothelial Function Biomarkers

The plasma, aortic and cardiac NO levels and the eNOS activity were measured by commercially available kits (Jiancheng Bioengineering, Nanjing, China) based on the manufacturer’s protocol.

### Measurement of Oxidative Stress Biomarkers

The heart and aorta tissues were homogenized in ice physiological saline using a homogenizer and then centrifuged at 5,000 r/min for 10 min. The supernatant was collected and the protein concentration was measured by the BCA method. The activities of SOD, GSH, and MDA levels in the aortic and cardiac homogenate were measured by corresponding kit (Jiancheng Bioengineering, Nanjing, China) based on the manufacturer’s protocols.

### Histopathological Analysis

The cardiac tissue was collected and washed by ice physiological saline, and then fixed by 10% formalin and embedded in paraffin. The pathological changes of the heart were examined using hematoxylin and eosin staining reagent. Cardiomyocyte injury and interstitial edema were evaluated for cardiac pathological score, in which the score was 0 for normal, 1 for mild, 2 for moderate, and 3 for severe damage.

### Cell Experiment

Rat H9C2 cells were purchased from China Infrastructure of Cell Line Resources (Chinese Academy of Medical Sciences) and cultured in Dulbecco’s modified Eagle’s medium, containing antibiotics and 10% fetal bovine serum at 37°C, 95% air, and 5% CO_2_. We changed the medium daily until the H9C2 cells were at 80–90% confluence. H9C2 cells (3 × 10^4^ cells/ml) were inoculated into a 96-well plate, and then added different concentrations of AS-IV (20, 40, and 80 μg/ml) in the absence or presence of L-NAME (1 mM) for 24 h. Then, cell Counting Kit-8 (CCK-8, Dojindo, Japan) was applied for measuring cell viability.

### Quantitative Real-Time Polymerase Chain Reaction

The whole RNA of H9C2 cells, heart, and aorta tissues was extracted by the TRIzol reagent (Invitrogen, United States ) following the manufacturer’s protocol. Then, the extracted total RNA was used for cDNA synthesis by the Prime Script RT reagent kit (Takara BioInc, Japan) according to the manufacturer’s protocol. qRT-PCR was carried out in the ABI StepOnePlus system (Applied Biosystems, United States ) using the Sybergreen™ reactions. The primers used in the present study were listed in supplementary file Supplementary Table S1. The results of mRNA were quantified using the delta Ct method and normalized to glyceraldehyde 3-phosphate dehydrogenase (GAPDH).

### Data Analysis

GraphPad Prism Version (version 7.0) software was used for all data analyses and all results were given as mean ± standard deviation (SD). Significant differences were analyzed by one-way analysis of variance (ANOVA) followed by the Mann Whitney test. A value of *p* < 0.05 was considered statistically significant.

## Results

### Targets Screening of AS-IV and Hypertensive Heart Disease

**FIGURE 1:**
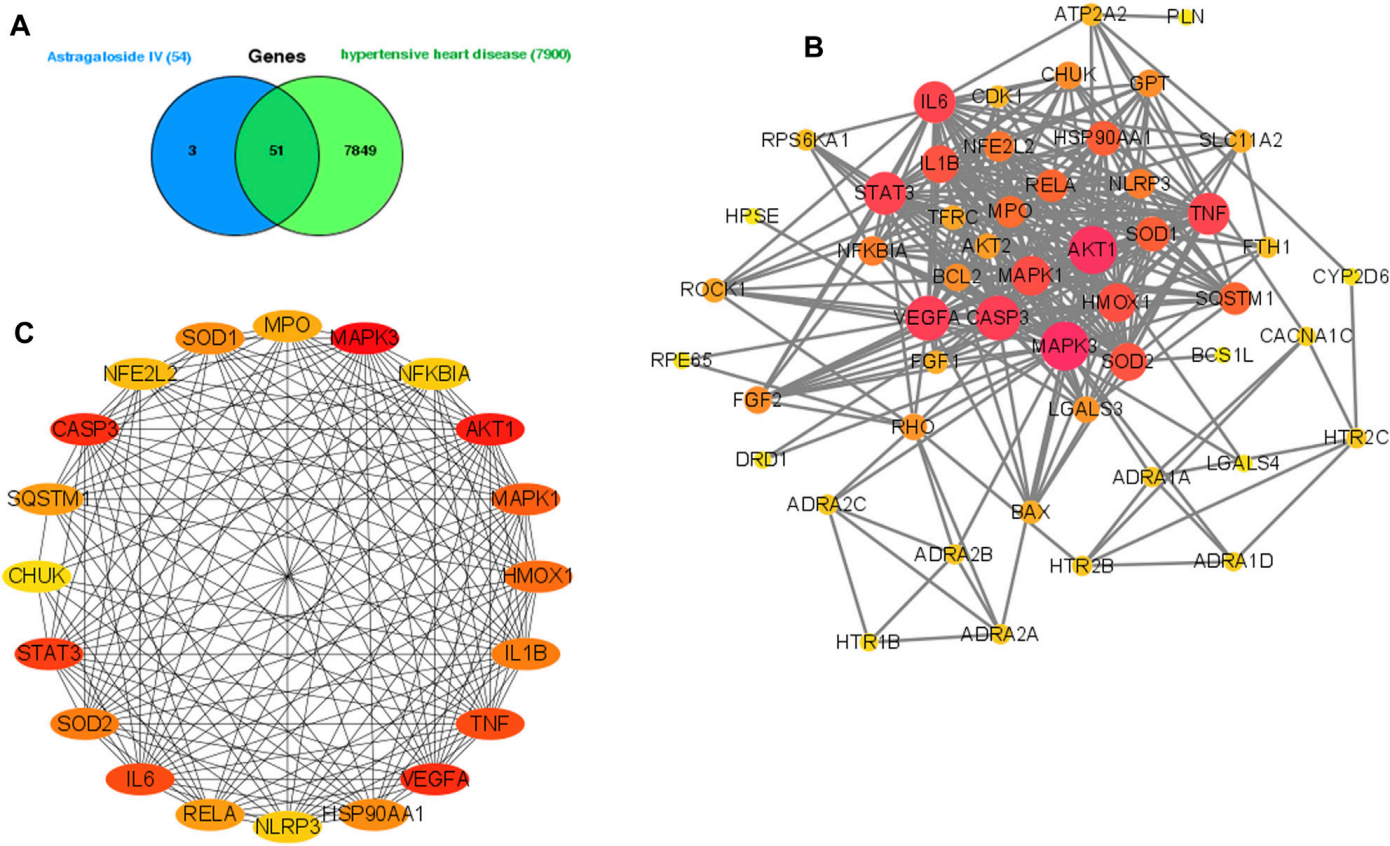
Analysis of the potential genes of AS-IV for the treatment of hypertensive heart disease. Venn diagram of 51 potential common genes **(A)**. Construction of a PPI network of those common genes. **(B)** PPI network construction of hub genes **(C)**, the darker color means the more important in the network.

As shown in Figure 1A, we collected potential genes of AS-IV from CTD, PubChem, and Swiss Target Prediction databases. Those genes were combined and we removed the overlap genes. Then, 54 genes associated with AS-IV were obtained. Besides, potential targets of hypertensive heart disease were predicted from the Genecards and CTD databases. We combined those potential genes and removed the overlap ones. Then, 7,900 hypertensive heart disease-associated genes were collected. Finally, 51 common genes were obtained as potential genes in the therapeutic effect of AS-IV against hypertensive heart disease.

### PPI Network Construction and Hub Genes Screening

As shown in Figure 1B, the string database was used to construct the PPI network of 51 common targets. Then, Cytoscape software was used to rearrange those 51 common genes based on the degree value, and the top 20 genes of high-node degree were selected as the hub genes (Figure 1C). Interestingly, we found the inflammation-related (TNF, IL-1β, and IL6) and oxidative stress-related (SOD1 and SOD2) genes in the hub genes.

### Enrichment Analysis of Common Genes

**FIGURE 2:**
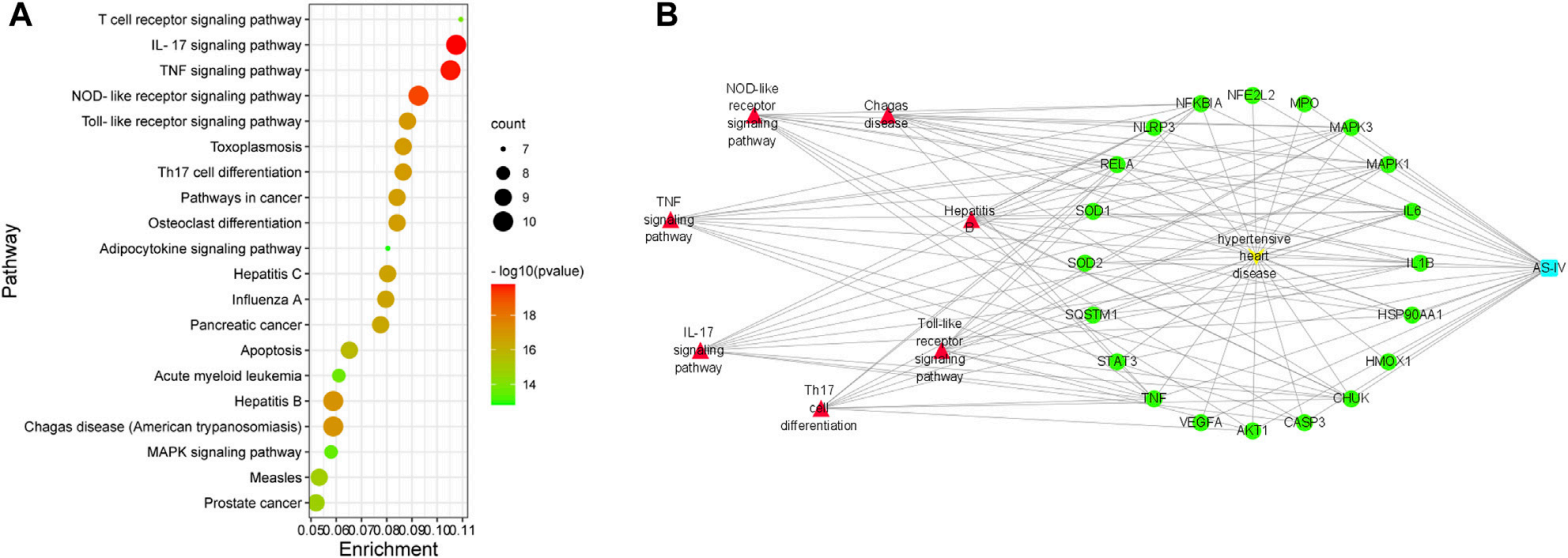
KEGG pathway enrichment analysis of top 20 genes. **(A)** Compound-targets-pathways-disease network: blue node is AS-IV. **(B)** Yellow node is hypertensive heart disease; green nodes represent 20 genes; red nodes represent seven potential signaling pathways; these lines indicate the interactions between them.

The KEGG enrichment analysis indicated how AS-IV acts on this pathway, thus exerting a therapeutic effect in hypertensive heart disease. In the present study, the top 20 hub genes were selected for enrichment analysis, and the top 20 signaling pathways were screened for further analysis based on *p*-Value. These signaling pathways were shown in Figure 2A. Among them, the IL-17 signaling pathway, TNF signaling pathway, NOD-like receptor signaling pathway, and Toll-like receptor signaling pathway as the top ones.

### Compound-Targets-Pathways-Disease Network

As shown in Figure 2B, a multidimensional network of “compound-targets-pathways-disease” was constructed by Cytoscape software, which included 29 nodes (1 compound, 20 genes, 7 signaling pathways, and 1 disease). The purple node is AS-IV; the yellow node is hypertensive heart disease; the green nodes represent 20 genes; the pink to red nodes represent 7 potential signaling pathways; these lines indicate the interactions between them. These findings indicated that the AS-IV could alleviate hypertension heart disease via regulating multi-targets and multi-signaling pathways.

### Effects of AS-IV on Cardiovascular Parameters and Cardiac Function in L-NAME-Treated Rats

**FIGURE 3:**
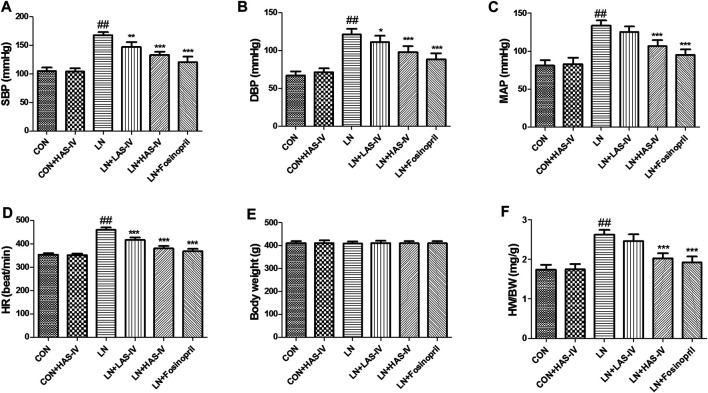
Effects of Astragaloside IV (AS-IV) on cardiovascular parameters in L-NAME-treated rats. The SBP **(A)**, DBP **(B)**, MAP **(C)**, HR **(D)**, body weight **(C)**, and HW/BW **(F)** was measured at the end of experiment. Results were showed as mean ± SD (*n* = 6 for each group). ##*p* < 0.001 versus CON group; *p < 0.01 versus LN group; **p < 0.01 versus LN group; ***p < 0.001 versus LN group.

**FIGURE 4:**
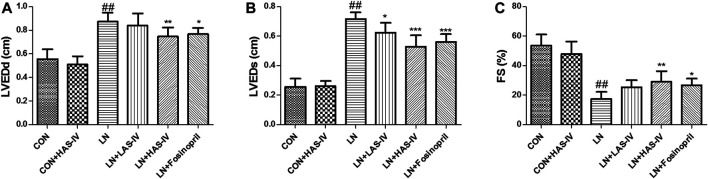
Effects of Astragaloside IV (AS-IV) on LVEDd **(A)**, LVEDs **(B)**, FS **(C)** values in L-NAME-treated rats. Results were showed as mean ± SD (*n* = 6 for each group). ##*p* < 0.001 versus CON group; *p < 0.01 versus LN group; **p < 0.01 versus LN group; ***p < 0.001 versus LN group.

As shown in Figure 3, administration of L-NAME for 5 weeks evoked a significant increase in SBP, DBP, MAP, HR, and HW/BW compared to the CON group. Treatment with a high dose of AS-IV (40 mg/kg) significantly decreased all these cardiovascular parameters in hypertensive heart disease rats. Fosinopril exerted similar effects as AS-IV (40 mg/kg). It’s worth noting that, a low dose of AS-IV (20 mg/kg) also decreased SBP, DBP, and HR in LN + LAS-IV group. Besides, our results indicated that the values of LVEDd and LVEDs in the LN group increased when compared with the CON group, accompanied by a significant decrease of FS (Figure 4). After AS-IV (40 mg/kg) or fosinopril treatment, LVEDd and LVEDs were decreased, while FS was increased, implying remarkable cardioprotection of AS-IV against L-NAME-induced hypertensive heart disease.

### Effects of AS-IV on Cardiac Marker Enzymes and Cardiac Hypertrophy in L-NAME-Treated Rats

**FIGURE 5:**
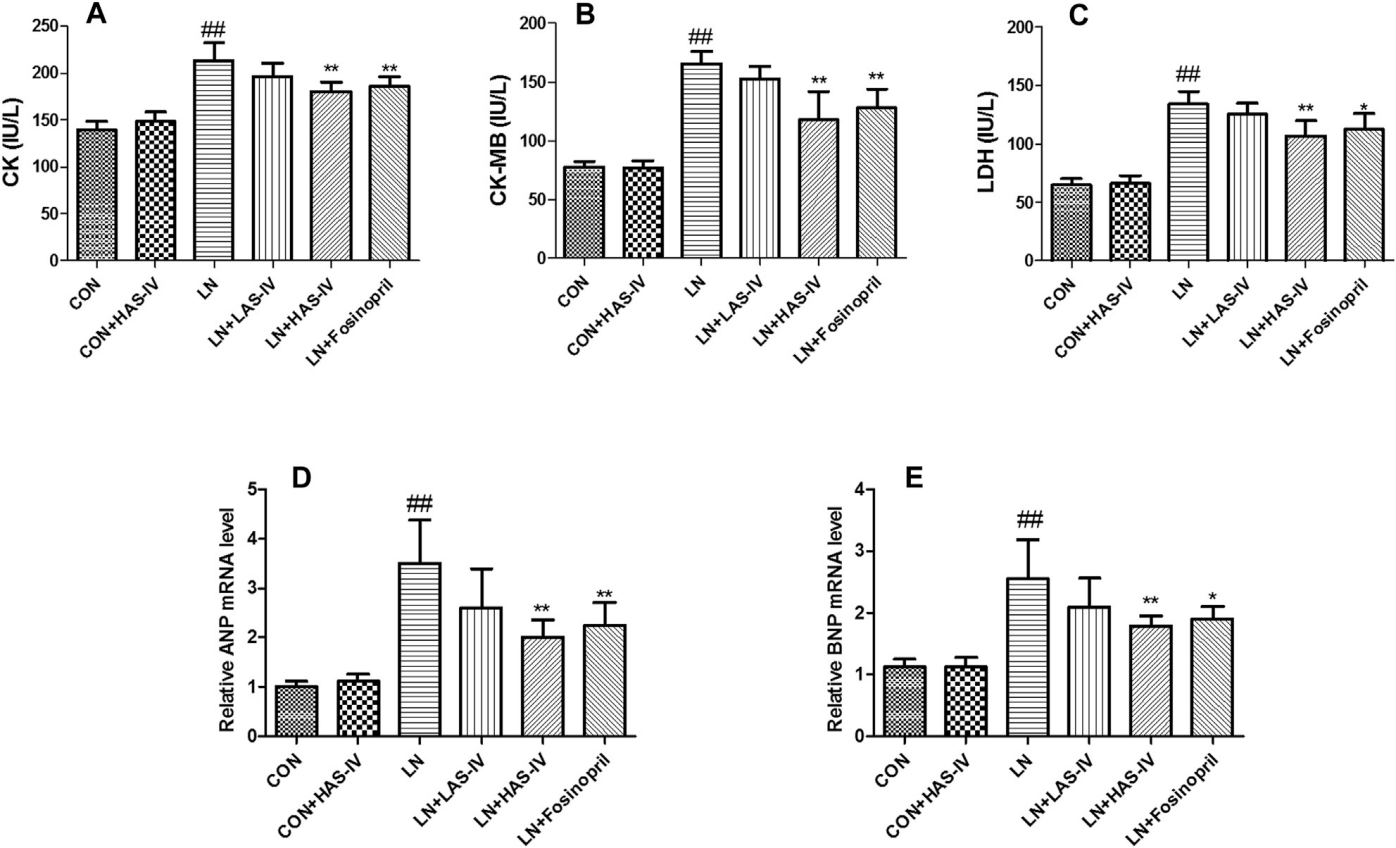
Effects of Astragaloside IV (AS-IV) on cardiac marker enzymes and cardiac hypertrophy in L-NAME-treated rats. The serum CK **(A)**, CK-MB **(B)**, LDH **(C)** activities were measured by commercially available kits. The cardiac tissue ANP **(D)**, BNP **(E)** mRNA expression levels were measured by qRT-PCR. Results were shown as mean ± SD (*n* = 6 for each group). ##*p* < 0.001 versus CON group; *p < 0.01 versus LN group; **p < 0.01 versus LN group; ***p < 0.001 versus LN group.

**FIGURE 6:**
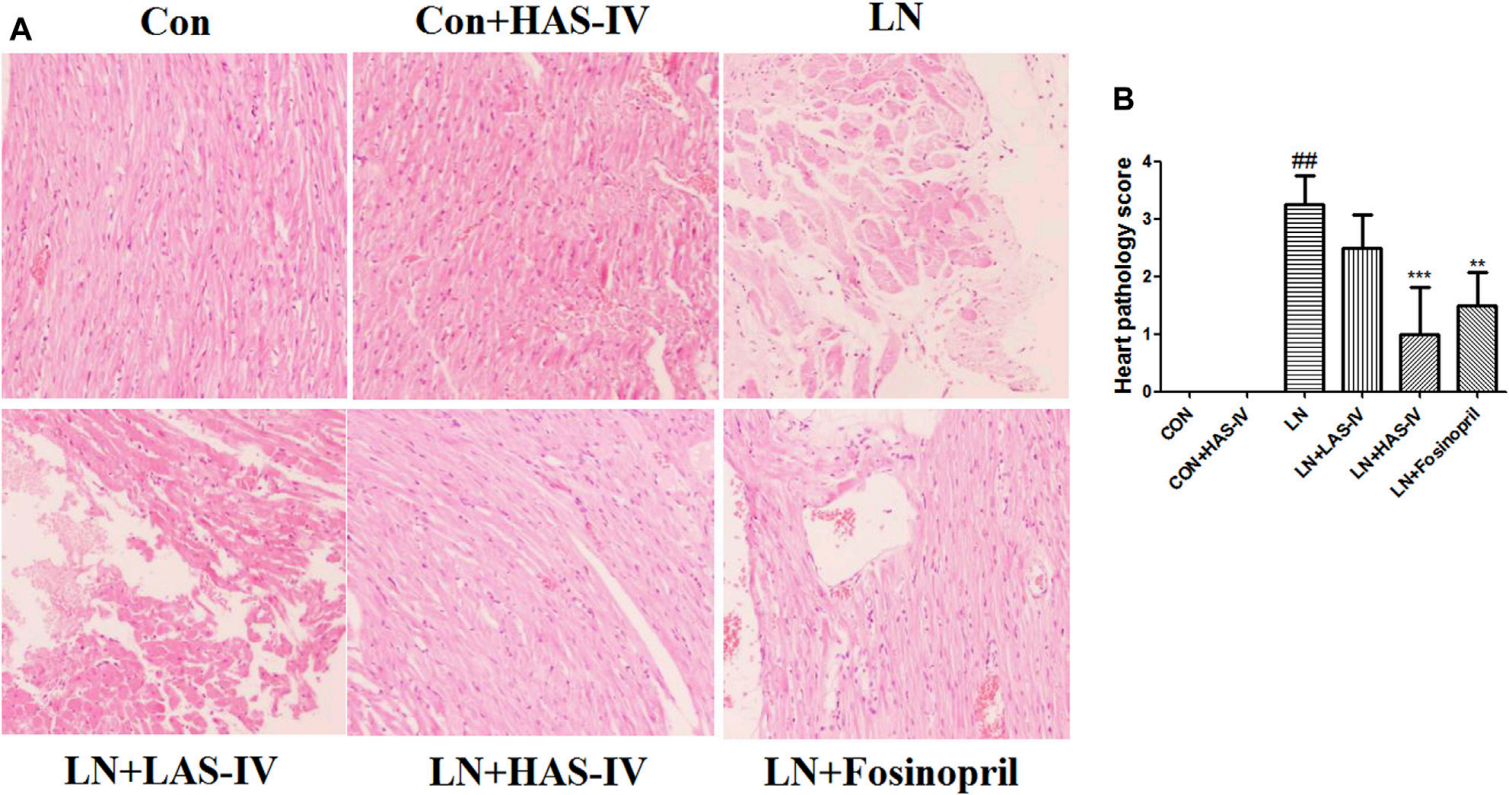
Effects of Astragaloside IV (AS-IV) on cardiac tissue histopathological changes in hypertensive rats. Results were shown as mean ± SD (*n* = 6 for each group). ##*p* < 0.001 versus CON group, **p < 0.01 versus LN group. Hematoxylin and eosin staining method was used to evaluate the cardiac tissue histopathological changes. **(A)** Cardiac pathological score **(B)**. Scale bar = 100 µm.

As shown in Figures 5A–C, after administration of L-NAME for 5 weeks, the activities of CK, CK-MB, and LDH were significantly increased in the LN group compared to the CON group. Treatment with a high dose of AS-IV (40 mg/kg) or fosinopril significantly reduced these cardiac marker enzymes activities in the model group. ANP and BNP are natriuretic peptides, play a major role in the regulation of cardiovascular, and as markers of myocyte hypertrophy (Gardner, 2003; Kohno et al., 1995). After administration of L-NAME for 5 weeks, the mRNA expression of ANP and BNP were significantly up-regulated in the LN group compared to the CON group (Figures 5D,E). Treatment with AS-IV (40 mg/kg) or fosinopril significantly reduced these cardiac hypertrophy markers expressions in the model group. Besides, hematoxylin-eosin (HE) staining was used to evaluate the pathologic features of cardiac tissue. Figure 6 showed an enlarged cross-sectional area of cardiomyocytes, cardiomyocyte injury, and interstitial edema in the cardiac sections of the LN group. Treatment with AS-IV (40 mg/kg) or fosinopril significantly alleviated these histopathological changes and decreased heart pathology score in the model group (Figures 6A,B). However, the low dose of AS-IV (20 mg/kg) has no effect on cardiac hypertrophy parameters and cardiac damage in L-NAME-treated rats. These findings revealed that the potential protective effects of AS-IV in the treatment of cardiac hypertrophy.

### Effects of AS-IV on Endothelial Function Biomarkers in L-NAME-Treated Rats

**FIGURE 7:**
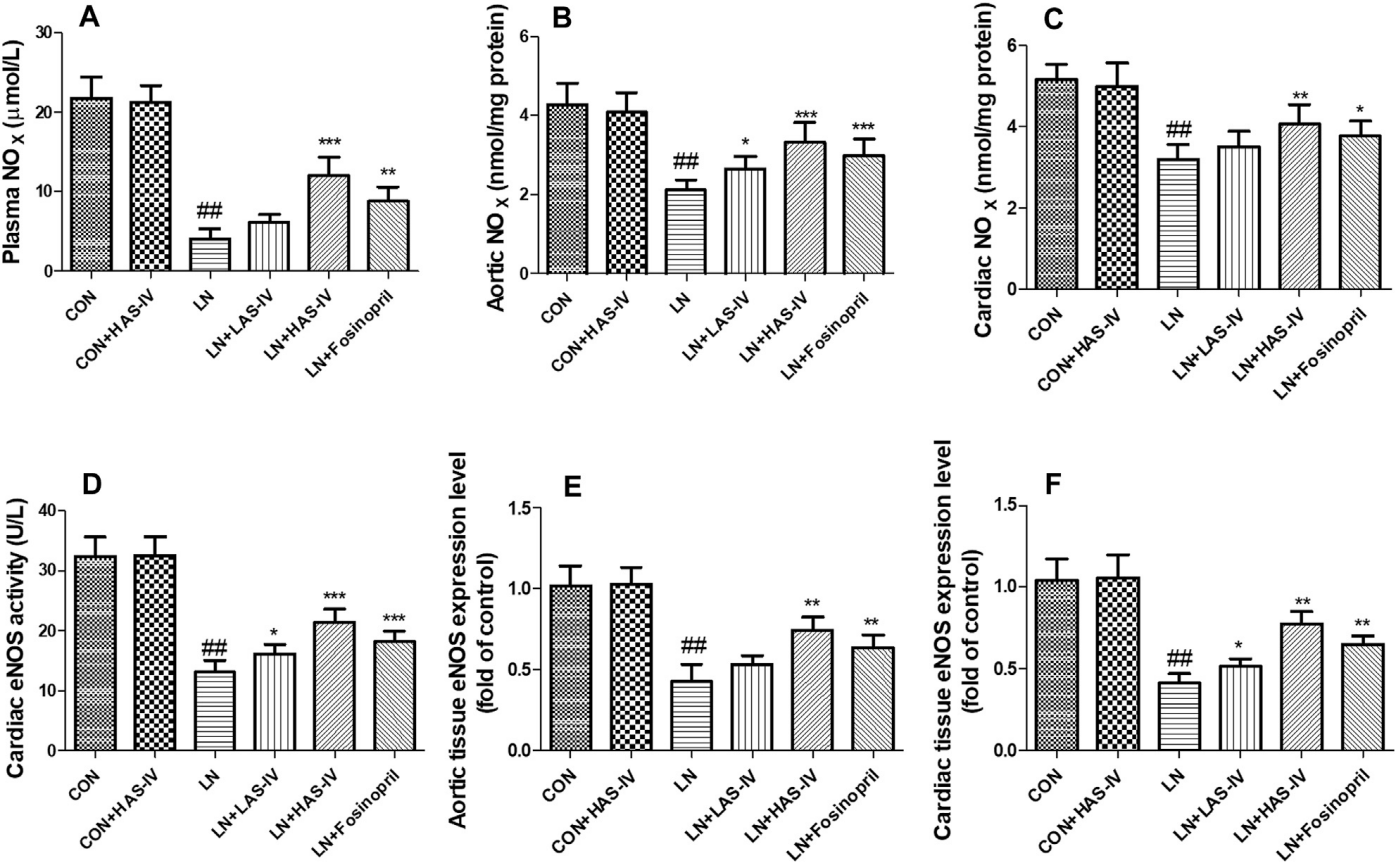
Effects of Astragaloside IV (AS-IV) on endothelial dysfunction in hypertensive rats evoked by L-NAME. The plasma NO_X_ **(A)**, aortic NO_X_ **(B)**, cardiac NO_X_ **(C)**, cardiac eNOS activity **(D)**, aortic eNOS mRNA expression **(E)**, cardiac eNOS mRNA expression **(F)** in CON, CON + HAS-IV, LN, LN + LAS-IV, LN + HAS-IV and LN + fosinopril groups. Results were showed as mean ± SD (*n* = 6 for each group). ##*p* < 0.001 versus CON group, *p < 0.01 versus LN group; **p < 0.01 versus LN group; ***p < 0.001 versus LN group.

Chronic administration of L-NAME for 5 weeks, the levels of NO_X_ and eNOS in plasma, heart, and aorta were significantly decreased in the LN group compared to the CON group (Figure 7). Treatment with AS-IV (40 mg/kg) or fosinopril significantly increased these endothelial function biomarkers in the hypertensive heart disease group.

### Effects of AS-IV on Inflammation Biomarkers in L-NAME-Treated Rats

**FIGURE 8:**
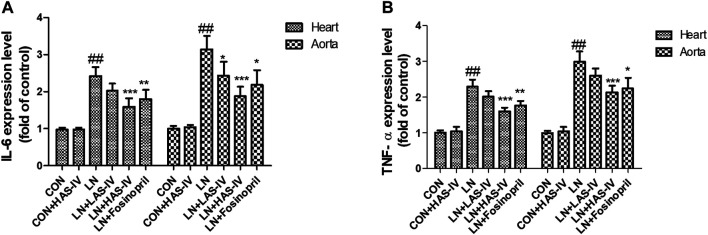
Effects of Astragaloside IV (AS-IV) on inflammation biomarkers in hypertensive rats evoked by L-NAME. The mRNA expression levels of IL-6 **(A)** and TNF-α **(B)** in CON, CON + HAS-IV, LN, LN + LAS-IV, LN + HAS-IV and LN + fosinopril groups. Results were shown as mean ± SD (*n* = 6 for each group). ##*p* < 0.001 versus CON group, *p < 0.01 versus LN group; **p < 0.01 versus LN group; ***p < 0.001 versus LN group.

Chronic administration of L-NAME for 5 weeks, the expression levels of IL-6 and TNF-α in the heart and aorta were significantly increased in the LN group compared to the CON group (Figure 8). Hypertensive heart disease rats treated with AS-IV (40 mg/kg) or fosinopril had significantly down-regulated IL-6 and TNF-α expression levels compared with the LN group.

### Effects of AS-IV on Oxidative Stress Biomarkers in L-NAME-Treated Rats

**FIGURE 9:**
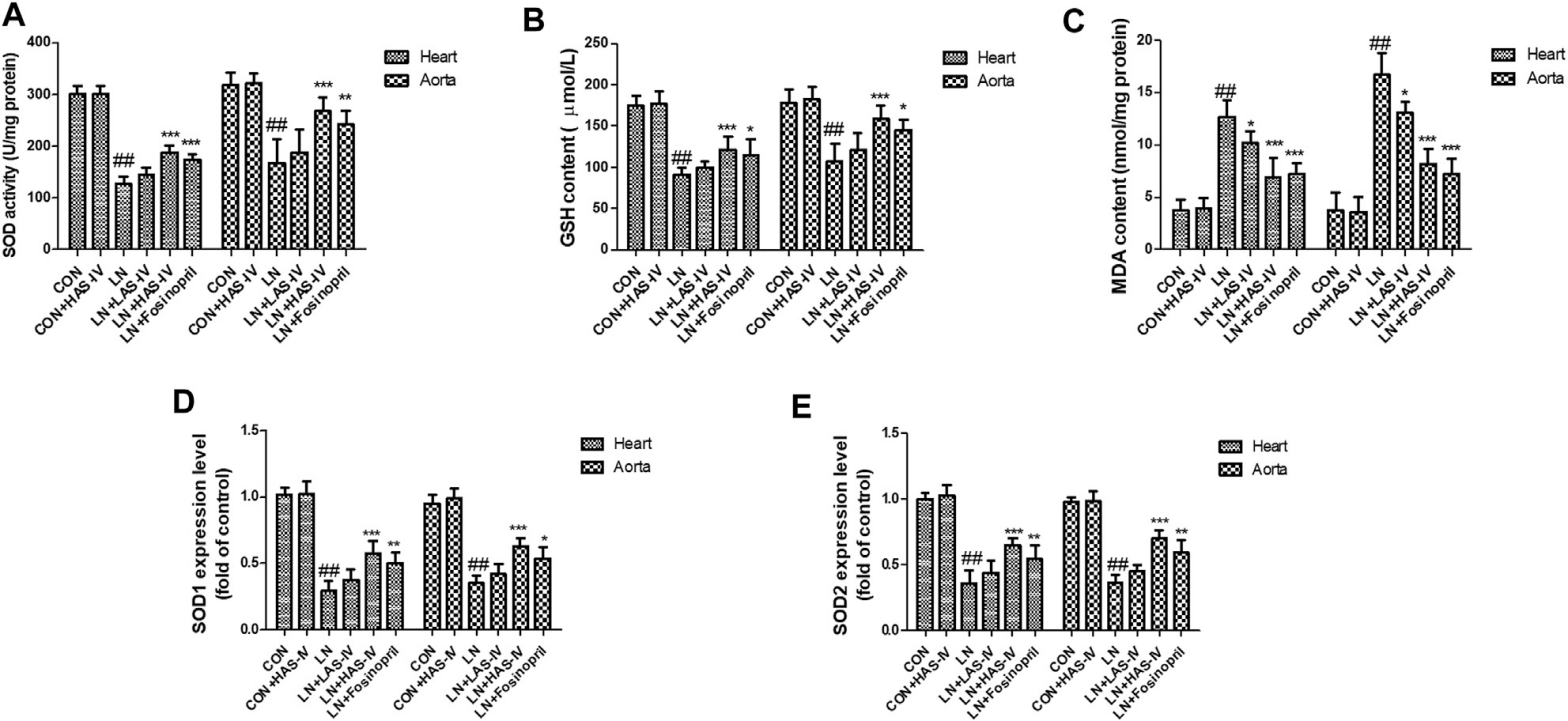
Effects of Astragaloside IV (AS-IV) on oxidative stress biomarkers in hypertensive rats evoked by L-NAME. The SOD activity **(A)**, GSH content **(B)**, MDA content **(C)**, SOD1 mRNA expression **(D)**, and SOD2 mRNA expression **(E)** in CON, CON + HAS-IV, LN, LN + LAS-IV, LN + HAS-IV and LN + fosinopril groups. Results were shown as mean ± SD (*n* = 6 for each group). ##*p* < 0.001 versus CON group, *p < 0.01 versus LN group; **p < 0.01 versus LN group; ***p < 0.001 versus LN group.

Chronic administration of L-NAME for 5 weeks, the activities of SOD and GSH in the heart and aorta were significantly decreased in the LN group compared to the CON group (Figures 9A,B). Besides, the levels of MDA were significantly increased in the LN group compared to the CON group (Figure 9C). Hypertensive heart disease rats treated with AS-IV (40 mg/kg) or fosinopril had significantly increased SOD and GSH activities and a significantly decreased MDA level compared with the LN group. It’s worth noting that, a low dose of AS-IV (20 mg/kg) also decreased MDA levels in LN + LAS-IV group. In addition, the qRT-PCR results indicated that AS-IV or fosinopril inhibited the down-regulation levels of SOD1 and SOD2 induced by L-NAME (Figure 9D,E).

### AS-IV Increased Cell Viability in L-NAME-Stimulated H9C2 Cells

**FIGURE 10:**
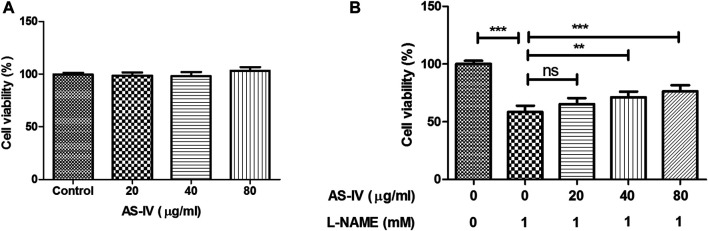
Effect of Astragaloside IV (AS-IV) on the cell viability in L-NAME-stimulated H9C2 cells. H9C2 cells were stimulated with different concentrations of AS-IV (20, 40, and 80 μg/ml) in the absence or presence of L-NAME (1 mM) for 24 h. Results were presented as mean ± standard deviation (SD). *n* = 4. **p < 0.01, ***p < 0.001.

As shown in Figure 10A, treatment with AS-IV (20–80 μg/ml) alone did not cause a decrease in cell viability of H9C2 cells. The H9C2 cells were exposed to L-NAME caused the decline of cell viability (Figure 10B). Treatment with AS-IV (40–80 μg/ml) improved cell viability in L-NAME-stimulated H9C2 cells.

### The Protective Effect of AS-IV Against L-NAME-Induced Inflammation and Oxidative Stress in H9C2 Cells

**FIGURE 11:**
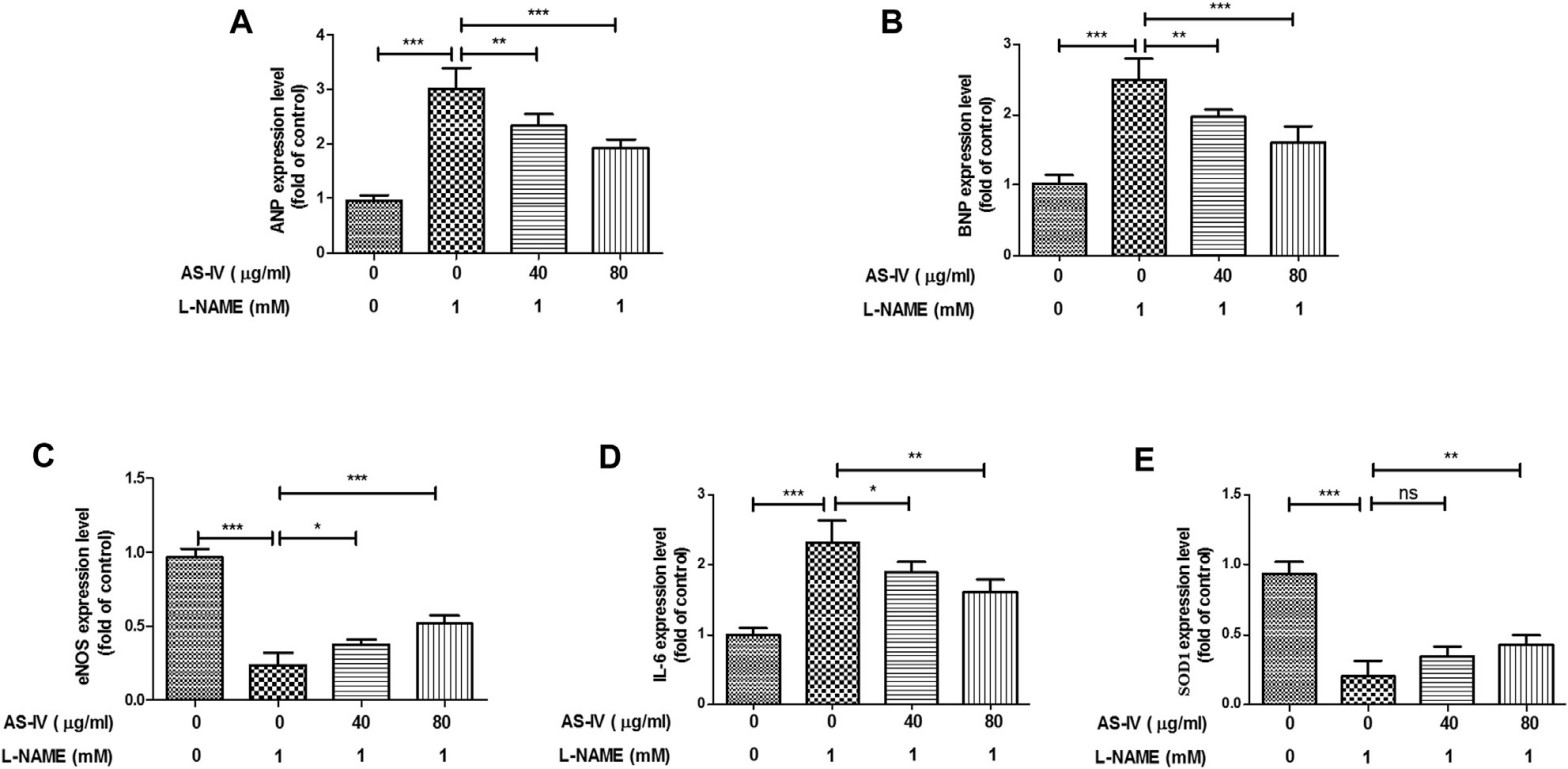
The protection effect of Astragaloside IV (AS-IV) against L-NAME-induced inflammation and oxidative stress in H9C2 cells. H9C2 cells were stimulated with different concentrations of AS-IV (40 and 80 μg/ml) in the absence or presence of L-NAME (1 mM) for 24 h mRNA expression of ANP **(A)**, ABP **(B)**, eNOS **(C)**, IL-6 **(D)**, and SOD1 **(E)** were measured by qRT-PCR. Results were presented as mean ± standard deviation (SD). *n* = 4. *p < 0.05, **p < 0.01, ***p < 0.001.

The cells experiment was performed to further validate the results of network pharmacology and animal experiment. As shown in Figure 11, the expression of ANP, BNP, and IL-6 was up-regulated in L-NAME-stimulated H9C2 cells. AS-IV significantly down-regulated the expressions of ANP, BNP, and IL-6. The expressions of eNOS and SOD1 were down-regulated in L-NAME-stimulated H9C2 cells. AS-IV significantly up-regulated the expressions of eNOS and SOD1.

## Discussion

Hypertensive heart disease is induced by chronic pressure overload and multiple mechanisms are involved in the progression of this disease (Slivnick and Lampert, 2019). Hyperphosphorylation of titin protein, microtubule disarray, and abnormal calcium handing are involved in this pathological process (Borbély et al., 2009; Shah et al., 2014). Therefore, the detailed etiology of hypertensive heart disease is not fully understood. Because there is no specific drug to treat hypertensive heart disease; therefore, developing novel agents is very necessary.

Over the past few decades, natural compounds extracted from herbs or Chinese herbal medicine have been one of the most primary resources for drug research and development, especially in the treatment and prevention of cardiovascular disease. AS-IV is the primary active compound extracted from *Astragalus membranaceus*. Previous reports have indicated that AS-IV exerts various protective activities in the brain, kidney, lung, and cardiovascular, and these pharmacological activities are related to multiple signaling pathways, such as Nrf2 antioxidant signaling pathways, NF-κB signaling pathway, and EGFR-Nrf2 signaling pathway (J. Zhang et al., 2020a). However, the underlying mechanism actions of AS-IV against hypertensive heart disease have not been fully understood. Network pharmacology integrates omics, network visualization, and other methods to establish the system network model based on the theory of systems biology (Li and Zhang, 2013). The complex relationship among drugs, diseases, targets, and pathways was revealed via network pharmacology analysis, which is of great significance for understanding the mechanism of action of traditional Chinese medicine, the basis of the medicinal substance, and the research and development of new drugs (Hao da and Xiao, 2014). In the present study, the network pharmacology was used to investigate the therapeutic effect of AS-IV in the treatment of hypertensive heart disease and an animal experiment was performed to verify our speculation.

First, we used network pharmacology and bioinformatics methods to construct a “compound-targets-pathways-disease” network. Our findings indicated that the targeted genes of AS-IV against hypertensive heart disease are involved in oxidative stress and inflammation, including SOD2, SOD1, IL-6, TNF, and IL-1β. Next, we investigated the antihypertensive and cardioprotective effects of AS-IV in L-NAME-induced hypertensive heart disease models. And our results revealed that these beneficial effects of AS-IV may be related to the down-regulation of IL-6 and TNF-α and up-regulation of SOD1 and SOD2.

At present, multiple mechanisms have been proposed for the etiology of hypertensive heart disease. One of the most important is the reduction of NO bioavailability or NO synthesis (Davel et al., 2011; Lee et al., 2016). Previous reports have indicated that the suppression of eNOS resulted in hypertension and high vascular resistance (Nyadjeu et al., 2013; Selamoglu Talas, 2014). Blockade NO synthesis in L-NAME-induced high blood pressure is related to the down-regulation of eNOS expression and elevation of MDA level (Fu et al., 2011; Jan-On et al., 2020). The L-NAME-induced hypertension heart disease model is similar to that of patients with vascular endothelial dysfunction, who also experience structural and functional cardiac dysfunction due to the loss of bioavailability of NO (Ndisang et al., 2014). *In vivo*, our findings showed that hypertension heart disease was induced by a decrease of NO_X_ level and an increase of oxidative stress. Administration of AS-IV decreased high blood pressure, increased eNOS expression as well as attenuated cardiac dysfunction in the hypertensive heart disease model. Meanwhile, MDA level and oxidative stress markers were also alleviated in the model group after AS-IV administration. Thus, antioxidant properties may be one of the mechanisms by which AS-IV inhibited the progression of hypertensive heart disease. Moreover, the administration of L-NAME also leads to cardiomyocytes injury that induces the secretion of cardiac enzymes (LDH, CK-MB, and CK). The previous report showed that chronic depletion of NO by L-NAME resulted in a significant increase in CK, CK-MB, and LDH levels (Kumar et al., 2014). Consistent with the previous report, our findings of cardiac enzyme measuring and H&E staining showed that the L-NAME administration caused the increase of cardiac enzymes (CK, CK-MB, and LDH) and cardiac structural abnormalities. In the present study, administration of AS-IV reversed those abnormalities induced by L-NAME, implying that AS-IV alleviated symptoms of hypertensive heart disease in L-NAME-treated rats.

Inflammation is a primary factor in the occurrence of disease (Nathan and Ding, 2010). The findings of the PPI network indicated that IL-6, TNF, IL-1β, and NFKBIA were predicted as the main genes of AS-IV against hypertensive heart disease and the KEGG pathways analysis showed that TNF signaling pathway was its key pathway. It has been reported that hypertensive caused chronic systemic inflammation, which stimulates the secretion of pro-inflammatory factors that lead to cardiac damage (Mouton et al., 2020). Chronic cardiac inflammation is involved in the aggravation of cardiac remodeling in hypertensive heart disease (Kai et al., 2009). AS-IV alleviates mechanical stress-induced myocardial hypertrophy *via* decreasing inflammation (T. Zhang et al., 2020b). AS-IV prevents lipopolysaccharide-induced gestational hypertension via the suppression of inflammatory responses (Tuerxun et al., 2021). Therefore, we speculated that AS-IV may alleviate systemic inflammation in hypertensive heart disease. In the present study, AS-IV down-regulated the expression of IL-6 and TNF and inhibited the progression of inflammation. This finding explained that AS-IV alleviated cardiac inflammation in hypertensive rats evoked by L-NAME.

Oxidative stress is also one of the pathogenic factors of hypertensive heart disease (Mi et al., 2019). Cardiac oxidative stress induced by excess reactive oxygen species has been indicated to be implicated in the occurrence and development of high blood pressure and pressure overload-induced cardiac damage (W. Zhao et al., 2008). It has been reported that oxidative injury was present in hypertensive myocardial tissue (Worou et al., 2011). Besides, the expression of SOD2 significantly changed in the hypertension-induced cardiac dysfunction (Koyanagi et al., 2008). Therefore, inhibition of oxidative stress is a novel method to treat hypertensive heart disease. AS-IV alleviates myocardial ischemia injury via suppressing reactive oxygen species burst and improving antioxidant potential (Luo et al., 2019). The findings of the PPI network indicated that SOD1 and SOD2 were predicted as the potential genes of AS-IV in the treatment of hypertensive heart disease. And the network pharmacological results were verified by *in vivo* and *in vitro* experiments. In the present study, we found that AS-IV up-regulated the expression of SOD1 and SOD2, improved the activities of SOD and GSH, and decreased the MDA level, indicating that AS-IV inhibited the progression of hypertensive heart disease via inhibition of oxidative stress.

## Conclusion

In the present report, the network pharmacology and experimental validation were performed to investigate the therapeutic effects of AS-IV against hypertensive heart disease. Our findings demonstrated that AS-IV prevents the progression of hypertensive heart disease *via* activation of eNOS and inhibition of oxidative stress. Our results not only provide a theoretical foundation for exploring the mechanism actions of AS-IV against hypertensive heart disease but also develop a promising treatment for hypertensive heart disease.

## Data Availability

The original contributions presented in the study are included in the article/Supplementary Files, further inquiries can be directed to the corresponding author.
