# Supplementary material for: The Mechanism Actions of Astragaloside IV Prevents the Progression of Hypertensive Heart Disease Based on Network Pharmacology and Experimental Pharmacology
Source: Front Pharmacol. 2021 Nov 5;12:755653. doi: 10.3389/fphar.2021.755653 (PMC8602690; doi:10.3389/fphar.2021.755653)
Supplement: Supplementary file 1 [file Table1.DOCX]

**Table S1.** Sequences of primers used quantitative real-time PCR.

| Gene | Forward primer (5' to 3') | Reverse primer (5' to 3') |
| --- | --- | --- |
| ANP | GGAGCCTGCGAAGGTCAA | TATCTTCGGTACCGGAAGCTGT |
| BNP | CAGAAGCTGCTGGAGCTGATAAG | TGTAGGGCCTTGGTCCTTTG |
| IL-6 | TCCTACCCCAACTTCCAATGCTC | TTGGATGGTCTTGGTCCTTAGCC |
| TNF-α | ATGGCATGGATCTCAAAGAC | CGGACTCCGTGATGTCTAAG |
| SOD1 | TGCTGAAGGGCGACGG | GTTCACCGCTTGCTTCTG |
| SOD2 | GAACCACAGGCCTTATTCCA | TCCAAGCAATTCAAGCCTCT |
| GAPDH | GACCCCTTCATTGACCTCAAC | CTTCTCCATGGTGGTGAAGA |
